# Exercise mode influences post‐exercise glucose sensitivity and insulin clearance in young, healthy males and females in a sex‐dependent manner: A randomized control trial

**DOI:** 10.14814/phy2.15354

**Published:** 2022-07-03

**Authors:** Kayleigh M. Beaudry, Julian C. Surdi, Andrea Mari, Michaela C. Devries

**Affiliations:** ^1^ Department of Kinesiology University of Waterloo Waterloo Canada; ^2^ Institute of Neuroscience, National Research Council Padova Italy

**Keywords:** high‐intensity interval exercise, low‐load high repetition resistance exercise, moderate intensity exercise, pancreas, sex comparison, 𝛽‐cell function

## Abstract

Type 2 diabetes (T2D) risk is lower in females than males. It has been reported that females have greater pancreatic 𝛽‐cell function than males, which may at least in part contribute to the T2D risk in females. 𝛽‐cell function is influenced by exercise training; however, previous trials comparing 𝛽‐cell function between the sexes have not included participants matched for training status. Furthermore, the acute effects of different modes of exercise on 𝛽‐cell function, and whether sex inherently influences these effects, are largely unexamined. Males and females (12/sex) completed a 120‐min oral glucose tolerance test (OGTT) at rest (CON) and following acute bouts of high‐intensity interval exercise (HIIE), moderate intensity continuous (MIC) exercise, and low‐load high‐repetition (LLHR) resistance exercise to assess whether sex inherently influences baseline and/or post‐exercise pancreatic function in the absence of pathology. We found no sex differences in basal pancreatic 𝛽‐cell function. Females had greater basal insulin clearance following MIC exercise compared to males (*p* = 0.01) and males tended to have a higher potentiation ratio following HIIE (*p* = 0.07). Females also had lower glucose sensitivity following MIC exercise compared to HIIE (*p* = 0.007) and LLHR (*p* = 0.003). Insulin clearance during the OGTT was greater following HIIE as compared with CON and MIC exercise (*p* = 0.02). 2‐H oral glucose insulin sensitivity was greater following LLHR compared to CON (*p* = 0.01). Acute bouts of different modes of exercise do not differentially influence 𝛽‐cell function but do influence insulin clearance and insulin sensitivity. Therefore, sex and exercise mode interact to differentially influence insulin clearance and glucose sensitivity.

## Graphical abstract

The findings of this study indicate that sex and exercise mode interact to differentially influence insulin clearance and glucose sensitivity. There were no sex differences in pancreatic function. However, exercise mode influenced insulin clearance and oral glucose insulin sensitivity. Furthermore, following an acute bout of moderate intensity continuous exercise, insulin clearance was greater in females than males. Additionally, in females only, glucose sensitivity and insulin clearance were higher following high intensity interval exercise compared with moderate intensity continuous exercise.

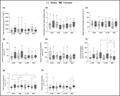

## 1 INTRODUCTION

The prevalence of type 2 diabetes (T2D) has risen dramatically in recent years to 422 million adults worldwide (Roglic, 2016). There is an inherent sex difference in T2D prevalence with 14 million more males having T2D than females (Aguiree et al., 2013). Furthermore, biological sex affects glucose homeostasis and parameters of insulin sensitivity such that women have a lower incidence of developing T2D later in life (Gannon et al., 2018; Nuutila et al., 1995; Varlamov et al., 2014). Interestingly, males are more frequently classified as having impaired fasting glucose, whereas females are classified as having impaired glucose tolerance due to elevated glucose concentrations at the end of an oral glucose tolerance test (OGTT) (Palmu et al., 2021; Pomerleau et al., 1999; Sicree et al., 2008), suggesting potential differences in disease pathogenesis. T2D is characterized by elevated blood glucose levels caused by an impairment in glucose tolerance from the development of insulin resistance (IR) and relative insulin deficiency (Mann et al., 2014). However, many individuals with IR maintain normal glycaemia due to compensatory increases in pancreatic insulin secretion (Malin et al., 2013; Malin et al., 2016). Thus, the preservation of pancreatic 𝛽‐cell function in IR may be fundamental to the prevention of T2D. Insulin secretion is reported to be higher in females than males in response to a given glucose load (Basu et al., 2006) and in response to an oral glucose tolerance test (Horie et al., 2018). However, in these trials aerobic fitness, which can markedly influence insulin sensitivity and 𝛽‐cell function (Dela et al., 2004; Slentz et al., 2009), was either not reported (Horie et al., 2018) or was reported but was ~16% higher in males when expressed relative to fat‐free mass as recommended when doing sex‐comparative research (Tarnopolsky, 2008). Thus, it is important to characterize whether sex inherently influences pancreatic 𝛽‐cell function in males and females matched for aerobic fitness and training history to further our understanding of how sex influences pancreatic 𝛽‐cell function.

Various modes of exercise training, including moderate‐intensity continuous training, high‐intensity interval training, and resistance training have been found to improve 𝛽‐cell function in several populations (Croymans et al., 2013; Madsen et al., 2015; Malin et al., 2018). However, the acute effects of exercise and the influence of exercise intensity and mode on 𝛽‐cell function are largely unexamined and are important to understand given that an acute bout of exercise can improve glycemic control for up to 72 h after the exercise bout. In one trial that examined the acute effects of moderate and high‐intensity exercise it was found that while both increased skeletal muscle disposition index, the early phase increase was greater with high‐intensity exercise (Malin et al., 2016), suggesting that exercise intensity may influence the acute effects of exercise on 𝛽‐cell function. Furthermore, it has been reported that the combination of aerobic and resistance training may induce a greater effect on 𝛽‐cell function (AbouAssi et al., 2015); however, this may have been due to a greater time spent exercising by participants in this group compared with the aerobic and resistance training alone groups. Low‐load, high‐repetition (LLHR) resistance exercise has emerged as a more aerobic form of resistance training that can induce similar gains in muscle mass and strength as high‐load, low‐repetition resistance training (Burd et al., 2010; Schoenfeld et al., 2015). Thus, the acute effects of a bout of LLHR resistance exercise matched for time to bouts of aerobic exercise of different intensities would allow us to compare the combined effects of aerobic and resistance exercise on 𝛽‐cell function to aerobic exercise alone without requiring additional exercise time.

Given the potential role of sex and exercise mode/intensity on pancreatic 𝛽‐cell function, the purpose of the present study was to investigate the effects of acute bouts of moderate intensity continuous (MIC) exercise, high‐intensity interval exercise (HIIE), and LLHR resistance exercise on indices of pancreatic 𝛽‐cell function including insulin secretion, rate sensitivity, and potentiation factor ratio, as well as insulin clearance and indices of insulin sensitivity in young recreationally active males and females. Additionally, we sought to examine whether sex influenced indices of pancreatic 𝛽‐cell function at rest and following exercise. We chose to study young, healthy males and females in order to determine whether there are inherent sex differences in the efficacy of different exercise modalities on 𝛽‐cell function in the absence of pathology. We hypothesized that both LLHR and HIIE would have a greater positive effect on pancreatic 𝛽‐cell function compared to MIC exercise. Additionally, we hypothesized that women would have greater pancreatic function at rest compared to males, but that this difference would disappear with exercise.

## 2 METHODS

### 2.1 Participants

Twenty‐four recreationally active young males (*n* = 12) and females (*n* = 12) took part in the study (Table 1). Participants were deemed recreationally active based on self‐reported habitual physical activity indicating that they participated in no more than three sessions of cardiovascular exercise or two sessions of resistance exercise per week. Additionally, participants were excluded if they had any chronic health conditions, were unable to complete a single exercise session, were unable to exercise as suggested by the get active questionnaire (GAQ) or had a BMI >27 kg/m^2^. Participants were instructed to maintain their habitual diet and physical activity throughout the trial. The study protocol was reviewed and received ethics clearance from the University of Waterloo Research Ethics Committee (ORE #22477). Prior to commencing the trial all participants provided written informed consent. The study conformed with all standards outlined by the Tri‐Council Policy Statements for Ethical Conduct for Research Involving Humans (TCPS 2) (Canadian Institutes of Health Research, 2018).

**TABLE 1:** Descriptive characteristics from male and female participants.

	Males	Females	*p* value
Age (years)	22 ± 1	21 ± 1	*p* = 0.45
Weight (kg)	76.6 ± 2.4	62.6 ± 3.1	*p* < 0.01*
Body mass index (kg/m^2^)	23.7 ± 0.8	22.9 ± 0.6	*p* = 0.45
Body fat (%)	20.9 ± 1.6	32.7 ± 1.3	*p* < 0.001*
Fat mass (kg)	15.4 ± 1.4	19.5 ± 1.2	*p* < 0.05*
Lean body mass (kg)	55.1 ± 2.2	37.6 ± 1.8	*p* < 0.001*
V̇O₂_peak_ (ml/kgBW/min)	43.1 ± 1.6	34.2 ± 1.4	*p* < 0.001*
V̇O₂_peak_ (ml/kgFFM/min)	59.5 ± 1.4	56.5 ± 1.7	*p* = 0.21

*Note*: All results are shown as mean ± SE, *n* = 12 males, and *n* = 12 females.

* Significance from non‐paired *t*‐test, significantly different with *p* value <0.05.

### 2.2 Experimental overview

**FIGURE 1:**
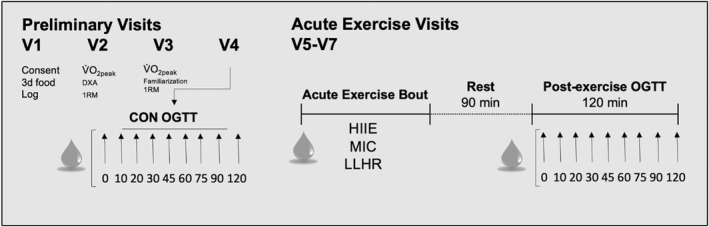
Protocol overview for preliminary visits (V1–V4), as well as the acute exercise visits (V5–V7). CON, control; HIIE, high‐intensity interval exercise; MIC, moderate intensity continuous exercise; LLHR; low‐load high repetition resistance exercise; OGTT, oral glucose tolerance test; V̇O₂_peak_, peak oxygen consumption; DXA, dual‐energy X‐ray absorptiometry; 1RM, 1 repetition max.

The experimental protocol consisted of four preliminary visits and three acute exercise sessions (Figure 1). The first preliminary visit included consent and anthropometric measurements (height, weight, BMI). At the end of this visit, participants were instructed on how to complete a 3‐day food log and were given a pedometer to track habitual physical activity. The second preliminary visit included a DXA scan (DXA, Hologic Discovery W with QDR APEX software version 4.5.3, Mississauga, ON, Canada) for the determination of body composition and an assessment of aerobic fitness (V̇O_2_ peak test) and maximal strength (1RM). The third visit included a familiarization session to the cycling bouts and a second 1RM assessment. Lastly, on the fourth visit, a control (CON) OGTT (75 g glucose, Trutol™️, Thermo Scientific, Waltham, Massachusetts, United States) was conducted. On the 5th–7th visit, participants completed acute bouts of HIIE, MIC exercise, and LLHR resistance exercise in randomized order. To allow for direct comparison of the effects of exercise intensity and mode, acute exercise sessions were designed to be completed in the same amount of time. Ninety minutes after the end of each acute exercise bout participants underwent a 120 min OGTT (75 g glucose, Trutol™️, Thermo Scientific). We conducted the post‐exercise OGTT 90 minutes after the cessation of exercise in order to examine the effects of exercise on pancreatic function in the insulin‐stimulated state as previous research has found that exercise‐induced GLUT4 at the sarcolemma reinternalizes 85 minutes after the cessation of exercise (Lauritzen, 2013). On the day before visits 4–7, participants consumed the same diet to minimize differences in fuel storage between assessments and to allow comparison of dietary intake between males and females. Given that menstrual phase can influence fuel metabolism during exercise (Devries et al., 2006) and insulin sensitivity (Trout et al., 2007), females were tested in the mid follicular phase of the menstrual cycle (day 5–9). There was a minimum of 3 days between study visits for all participants. As it is imperative to control for the menstrual cycle phase, females were tested across 2–3 menstrual cycles in order to allow for at least 3 days between acute exercise sessions while still testing females in the follicular phase of their cycle. The overall study schematic is found in Figure 1.

### 2.3 V̇O₂_peak_ testing

To appropriately match males and females for fitness level and to determine the work rate corresponding to 90% HR_max_ for the acute HIIE trial and 65% V̇O₂_peak_ for the MIC exercise bout, participants underwent an incremental cycling test to volitional fatigue on an electronically braked cycle ergometer (Ergoselect 100, ergoline GmbH, Germany) using an online gas collection system (Vmax encore CPET Systems, Vyaire medical, Chicago) to measure oxygen consumption. Participants began with a warmup at 50 W for 2mins and thereafter the intensity was increased by 1 W every 2 s until volitional fatigue or the point at which pedal cadence fell below 50 rpm. In order to ensure that males and females were equally trained (Tarnopolsky, 2008) V̇O₂_peak_ was expressed relative to fat‐free mass. Participants returned to the lab at least 24‐h after the V̇O₂_peak_ testing and cycled at the approximate wattage of the HIIE and MIC exercise bouts to ensure that the work rate was appropriate to achieve the target HR of 90% HR_max_ and 65% V̇O₂_peak_, respectively.

### 2.4 1‐RM testing

To determine the workload that corresponded to 30% 1 repetition max (1RM) for each exercise to be completed during the LLHR trial participants completed two separate 1RM (strength) testing sessions. Participants completed 1RM testing for chest press (CP, Technogym, Cesena, Italy), shoulder press (SP, Technogym, Cesena, Italy), lat Pulldown (LatP, Technogym, Cesena, Italy), leg press (LP, Technogym, Cesena, Italy), knee extension (KE, Technogym, Cesena, Italy), and hamstring curl (HC, Technogym, Cesena, Italy). The same investigators administered all strength testing. After a brief general warm‐up (5 min, cycling), a specific warmup of the given exercise was performed at approximately 50% of the participants estimated 1RM based on habitual 10RM. The load was progressively increased by ~10%–20% for each attempt until a true 1RM was reached. Three minutes of rest was given between each attempt. A successful attempt required the participant to move the load throughout the full range of motion for the exercise with correct form. The higher load of the two separate 1RM tests was used to determine 30% of participants 1RM for the LLHR bout.

### 2.5 Control OGTT and acute exercise trials

On the morning of the CON OGTT and acute exercise sessions participants arrived at the laboratory following a 12‐h overnight fast and having refrained from alcohol for 24‐h and moderate‐vigorous physical activity for 72‐h prior to each testing day. Upon arrival in the laboratory, participants rested quietly and an indwelling catheter was inserted into a prominent forearm vein and a fasted blood sample was taken. Participants then completed either the CON OGTT (described below) or the appropriate acute exercise bout (described below). For the acute exercise sessions, the participants sat quietly for 90 min after the exercise session and then completed the post‐exercise OGTT.

### 2.6 Oral glucose tolerance tests

Participants completed one CON OGTT and three post‐exercise OGTTs. After the fasted blood sample was taken (*t* = 0) participants then consumed 75 grams of glucose (Trutol™️, Thermo Scientific, Waltham, Massachusetts, United States) and blood samples were taken at 10, 20, 30, 45, 60, 75, 90, and 120 min after drink consumption. Heparinized plasma samples were analyzed for insulin and c‐peptide concentrations using radioimmunoassay (RIA) kits (#HI‐14K and HCP‐20K) according to manufacturer's instruction (Millipore Sigma). Blood glucose concentration was analyzed using a spectrophotometric glucose assay using PGO enzyme preparation (Sigma‐Aldrich).

### 2.7 High‐intensity interval exercise bout

The acute HIIE bout started with a 5‐min warm‐up at 50 W, followed by 10 intervals of 60 s at 90% HR_max_ interspersed with 60 s at low‐intensity (50 W) and ended with a 5 min cool down at 50 W. Heart rate was recorded every minute throughout the exercise bout. This work rate was chosen to represent a typical “low volume” interval exercise session that constitutes <10 mins of intense exercise and has been previously shown to induce favorable changes in insulin sensitivity (Dela et al., 2019; DiPietro et al., 2006; Gibala et al., 2014; Hood et al., 2011; Sogaard et al., 2017).

### 2.8 Moderate‐intensity continuous exercise bout

The MIC exercise bout consisted of participants performing a 30‐minute bike ride on a cycle ergometer (Ergoselect 100, ergoline GmbH, Germany) at 65% of their predetermined V̇O_2peak_. Heart rate was recorded every 3 min throughout the exercise bout. This intensity was chosen to represent a typically aerobic exercise intervention. MIC exercise has been well established for its beneficial effects on glucose handling, insulin sensitivity, and 𝛽‐cell function (Tessier et al., 2000; Yang et al., 2014).

### 2.9 Low‐Load high repetition exercise bout

The LLHR resistance exercise bout consisted of three cycles of a whole‐body circuit consisting of CP, KE, LatP, HC, SP, and LP exercises at 30% 1‐RM for 20–25 repetitions with the last set of each exercise continuing to volitional failure. Each exercise was separated by 30 seconds of rest and each round of the circuit was separated by 2 min of rest. Heart rate was recorded at the end of each set of exercises throughout the exercise bout. LLHR exercise has been previously demonstrated to elicit the same beneficial adaptations on myofibrillar protein synthesis as high‐load resistance exercise (Burd et al., 2010), but is much more cardiovascular in nature and therefore allowed us to examine the acute effects of combined aerobic and resistance exercise on 𝛽‐cell function.

### 2.10 Calculations

Indicators of pancreatic function were calculated using standardized equations. Insulin secretion at 5 mmol/L glucose concentration, which is the insulin secretion at a glucose concentration similar to fasting values, was calculated from the 𝛽‐cell dose–response as previously described (Utzschneider et al., 2007). Rate sensitivity, which is a parameter characterizing early insulin secretion and is a raw marker of first‐phase insulin secretion, was calculated as previously described (Utzschneider et al., 2007). Potentiation factor, which quantifies the time‐dependent increase in insulin secretion during the OGTT, was calculated as previously described (Utzschneider et al., 2007). The potentiation ratio, which is the ratio between the potentiation factor value at 2 h and that at time 0, was calculated as previously described (Mari et al., 2013). Basal insulin clearance is the insulin clearance calculated from basal values as (insulin secretion)/(insulin concentration) (Utzschneider et al., 2007). OGTT insulin clearance is the mean insulin clearance during the OGTT, calculated as (mean insulin secretion)/(mean insulin concentration) (Utzschneider et al., 2007). Glucose sensitivity, which is the slope of the 𝛽‐cell dose–response and is the main parameter characterizing 𝛽‐cell function, was calculated as previously described (Mari et al., 2010). The Matsuda index was calculated based on the method of Matsuda and DeFronzo (1999). The Matsuda index was included as it is a commonly used indicator of whole‐body insulin sensitivity that is reflective of both muscle and hepatic insulin sensitivity and is derived using glucose and insulin values in the fasted state and during the OGTT (Patarrão et al., 2014). The Stumvoll insulin sensitivity index was calculated based on the method of (Stumvoll et al., 2000). The Stumvoll insulin sensitivity index was included as it is based on both glucose and insulin concentrations during the OGTT and also considers age and BMI (Patarrão et al., 2014; Stumvoll et al., 2000). The 2‐h oral glucose insulin sensitivity (OGIS) was calculated according to the method of (Mari et al., 2001). The 2‐h OGIS is an estimate of glucose clearance during an OGTT and is validated against the hyperinsulinemic‐euglycemic clamp (Mari et al., 2001). The 2‐h OGIS is expressed as ml/min per square meter of body surface area and therefore is suitable to detect differences between males and females whose body surface areas will inherently differ based on sex differences in height, weight, and adiposity (Mari et al., 2001).

### 2.11 Statistical analysis

All statistical analyses were conducted using SPSS (version 25, IBM). Differences in baseline characteristics between males and females were assessed using a non‐paired *t*‐test. Additionally, as it was important to determine whether sex influenced pancreatic function, insulin clearance, and/or insulin sensitivity basally and during the OGTT independent of exercise, we also examined sex differences during the CON OGTT using a non‐paired *t*‐test. Two‐way mixed model ANOVA with sex as the between variable and trial (4 levels, CON/Exercise sessions) as the within variable was used to determine the effects of sex and trial on all measures during the OGTT. Data sets were assessed for normality using the Kolmogorov–Smirnov test and were found to be not normally distributed. Thus, values were log‐transformed prior to undergoing statistical analyses using ANOVA. Post‐hoc analyses were conducted using a Tukey's HSD test where appropriate. Significance was set at *p* ≤ 0.05. Partial eta‐squared (*η*_p_^2^) values were calculated to estimate the effect sizes (small 0.04, medium 0.25, large 0.64) for main effects and interactions where necessary (Ferguson, 2009). Cohen's 𝘥 values were calculated to estimate effect sizes (small 0.2, medium 0.5, large 0.8) for *t*‐tests and post hoc comparisons where necessary (Cohen, 1988). All data are presented as means ± SEM for *n* = 12 in each group as pre log‐transformed data. All graphs were created using GraphPad Prism (GraphPad Software Inc.).

## 3 RESULTS

### 3.1 Descriptive characteristics

Descriptive characteristics for the 24 participants who participated in the study are presented in Table 1. There were significant differences in weight, %BF, lean body mass, and V̇O₂_peak_ between males and females, which was expected. Once V̇O₂_peak_ was adjusted based on fat free mass rather than body weight, there was no significant difference between males and females, indicating that our males and females were equally trained.

### 3.2 Habitual dietary intake

Average dietary intake of all participants from the 3‐day food log are presented in Table 2. Overall males consumed more calories per day and grams per day of protein, fat, and carbohydrates compared to females (*p* < 0.01). However, when expressed as percent of total daily caloric intake, males and females ate similar percentages of protein, fat, and carbohydrates. There was no difference in relative protein consumption (g/kgBW/d) between males and females. Diets did not differ between males and females or between trials for the day before the CON OGTT and acute exercise sessions (data not shown).

**TABLE 2:** Three‐day diet data for males and females

	Males	Females	*p* value
Kcal	2008 ± 200	1477 ± 61	*p* < 0.01*
Protein			
g	96.4 ± 7.8	65.0 ± 3.4	*p* < 0.01*
g/kgBW/d	1.3 ± 0.1	1.1 ± 0.1	*p* = 0.10
% Of Kcals	18.8 ± 1.2	17.4 ± 0.8	*p* = 0.35
Total fat			
g	79.1 ± 5.6	56.5 ± 3.6	*p* < 0.01*
% Of Kcals	34.8 ± 1.4	33.1 ± 1.4	*p* = 0.38
Carbohydrate			
g	234.0 ± 15.8	185.6 ± 9.2	*p* = 0.01*
% Of Kcals	46.3 ± 1.7	49.5 ± 1.5	*p* = 0.16

*Note*: All results are shown as mean ± SEM, *n* = 12 males and *n* = 12 females.

* Significance from non‐paired *t*‐test, significantly different with *p* value <0.05.

### 3.3 Heart rate data

**FIGURE 2:**
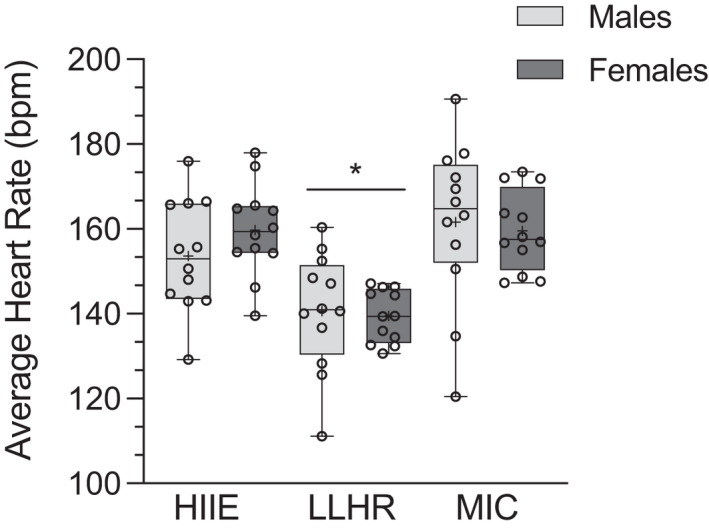
Average heart rate during the acute exercise bouts in males (*n* = 12) and females (*n* = 12). *Denotes significantly different compared to HIIE (*p* < 0.001, *d* = 1.5) and MIC (*p* < 0.001, *d* = 1.6) from mixed measures ANOVA.

Average heart rate (HR) during the acute exercise bouts is shown in Figure 2. There was a main effect of trial on HR (*p* < 0.001, *η*_p_^2^ = 0.40) whereby average HR during LLHR was lower than both the HIIE and MIC exercise bouts. There was no main effect of sex (*p* = 0.77, *η*_p_^2^ = 0.00) and no trial × sex interaction (*p* = 0.26, *η*_p_^2^ = 0.06).

### 3.4 Baseline sex differences in pancreatic 𝛽‐cell function, insulin clearance, and insulin sensitivity

**FIGURE 3:**
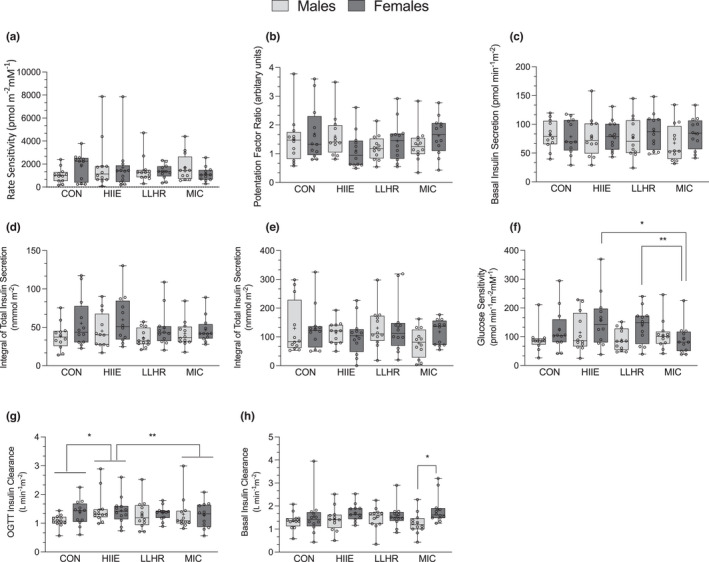
Measures of pancreatic function and insulin clearance in males (*n* = 12) and females (*n* = 12) at rest and following different modes of exercise. (a) Rate sensitivity; (b) potentiation factor; (c) basal insulin secretion; (d) integral of total insulin secretion; (e) insulin secretion at 5 mmol/L glucose; (f) glucose sensitivity, *higher than MIC exercise in females (*p* = 0.007, *d* = 0.81), **higher than MIC exercise in females (*p* = 0.003, *d* = 0.99); (g) OGTT insulin clearance, *greater than CON (*p* = 0.003, *d* = 0.62) and ** greater than MIC exercise (*p* = 0.02, *d* = 0.51); (h) basal insulin clearance, *higher following MIC exercise in females vs. males (*p* = 0.01, *d* = 1.2). Significance determined from mixed model ANOVA.

Baseline differences in pancreatic 𝛽‐cell function between males and females are shown in Table 3. There were no differences between males and females for basal glucose, mean glucose, 2‐h glucose during the OGTT, basal insulin, mean insulin during the OGTT, basal insulin secretion, insulin secretion at 5 mmol/L, glucose sensitivity, integral of total insulin secretion, rate sensitivity, potentiation factor ratio, basal insulin clearance or insulin clearance during the CON OGTT (*p* > 0.07, *d* < 0.79; Table 3 and Figure 3). There were no differences in markers of insulin sensitivity such as 2 h OGIS, Stumvoll or Matsuda insulin sensitivity index at baseline between males and females (*p* > 0.42, *d* < 0.33; Table 3).

**TABLE 3:** Sex differences in pancreatic function, insulin clearance and insulin sensitivity during the control OGTT.

	BASELINE		*p* value	*Cohen's d*
	Males	Females		
Basal glucose (mmol/L)	5.4 ± 0.2	5.1 ± 0.7	0.43	0.33
Mean glucose (mmol/L)	6.7 ± 1.2	7.3 ± 1.5	0.36	0.38
2‐h glucose (mmol/L)	6.0 ± 0.3	6.8 ± 0.4	0.15	0.61
Basal insulin (pmol/L)	61.9 ± 4.2	57.4 ± 6.7	0.41	0.34
Mean insulin (pmol/L)	289.2 ± 30.4	343.0 ± 47.4	0.45	0.31
Basal insulin secretion (pmol min^−1^ m^−2^)	81.9 ± 7.3	78.3 ± 8.6	0.65	0.19
Insulin secretion at 5 mmol/L (pmol min^−1^ m^−2^)	129.6 ± 27.0	119.3 ± 24.9	0.34	0.39
Glucose sensitivity (pmol min^−1^ m^−2^ mM^−1^)	89.1 ± 12.5	122.3 ± 21.0	0.26	0.49
Integral of total insulin secretion (nmol m^−2^)	38.2 ± 5.0	55.3 ± 9.4	0.13	0.63
Rate sensitivity (pmol m^−2^ mM^−1^)	949.7 ± 710.9	1808.7 ± 1150.7	0.27	0.46
Potentiation factor ratio (dimensionless)	1.49 ± 0.9	1.70 ± 1.0	0.57	0.24
Basal insulin clearance (L min^−1^ m^−2^)	1.4 ± 0.1	1.5 ± 0.2	0.80	0.10
OGTT insulin clearance (L min^−1^ m^−2^)	1.5 ± 0.2	1.4 ± 0.1	0.12	0.66
2 h OGIS (mL min^−1^ m^−2^)	408.8 ± 14.6	411.6 ± 16.5	0.92	0.04
Stumvoll (mL min^−1^ kg^−1^)	9.3 ± 0.4	8.6 ± 0.4	0.51	0.28
Matsuda (AU)	5.1 ± 0.6	5.2 ± 0.7	0.91	0.05

*Note*: All results are shown as mean ± SEM, *n* = 12 males and *n* = 12 females. *p*‐values based on log transformed data.

Significance *p* < 0.05 from non‐paired *t*‐test.

### 3.5 Post‐exercise pancreatic 𝛽‐cell function and insulin clearance

There was no effect of sex (*p* = 0.74, *η*_p_^2^ = 0.01), trial (*p* = 0.61, *η*_p_^2^ = 0.02) or sex × trial interaction (*p* = 0.22, *η*_p_^2^ = 0.07) on rate sensitivity (Figure 3a). Additionally, there was no effect of sex (*p* = 0.93, *η*_p_^2^ = 0.00) or trial (*p* = 0.59, *η*_p_^2^ = 0.03, Figure 3b) for potentiation factor ratio (Figure 3b); however, there was a trend for a sex × trial interaction (*p* = 0.07, *η*_p_^2^ = 0.10) indicating that males tended to have a significantly greater potentiation factor ratio following HIIE compared to females. There was no main effect of sex (*p* = 0.28, *η*_p_^2^ = 0.05) or trial (*p* = 0.11, *η*_p_^2^ = 0.09) on glucose sensitivity. There was a significant sex × trial interaction (*p* = 0.04, *η*_p_^2^ = 0.12) indicating that glucose sensitivity was significantly lower following MIC exercise compared to HIIE and LLHR in females (Figure 3f). Additionally, there was a significant main effect of trial on basal glucose at the start of the post‐exercise OGTT (*p* = 0.04, *η*_p_^2^ = 0.125) such that following LLHR basal glucose was significantly lower than CON (Table 4). There was no effect of sex (*p* = 0.97, *η*_p_^2^ ≥ 0.00) or sex × trial interaction (*p* = 0.10, *η*_p_^2^ = 0.09) on basal glucose. There was also a significant effect of trial on mean glucose (*p* = 0.03, *η*_p_^2^ = 0.14) whereby compared to both CON and LLHR, mean glucose during the OGTT following the MIC exercise bout was higher (Table 4). There was no effect of sex or sex × trial interaction for mean glucose during the OGTT (*p* > 0.19, *η*_p_^2^ ≥ 0.01; Table 4). There was no effect of sex (*p* ≥ 0.16, *η*_p_^2^ ≥ 0.01), trial (*p* ≥ 0.09, *η*_p_^2^ ≥ 0.01) or sex × trial interaction (*p* ≥ 0.09, *η*_p_^2^ ≥ 0.01) for 2‐h glucose during the OGTT, basal insulin, mean insulin, basal insulin secretion, insulin secretion at 5 mmol/L from the 𝛽‐cell dose response or integral of insulin secretion (Figure 3 and Table 4).

**TABLE 4:** Basal and mean glucose and insulin values during the control and post‐exercise OGTT.

Variable	Acute exercise bouts								*p* values		
	CON		HIIE		MIC		LLHR		S	T	S × T
	Males	Females	Males	Females	Males	Females	Males	Females			
Basal glucose (mmol/L)	5.6 ± 0.2	5.1 ± 0.2	5.1 ± 0.1	5.0 ± 0.2	5.0 ± 0.2	5.1 ± 0.1	4.8 ± 0.1	5.1 ± 0.2	0.97	0.04^a^	0.10
Mean glucose (mmol/L)	6.7 ± 0.3	7.3 ± 0.4	6.9 ± 0.3	7.5 ± 0.3	7.0 ± 0.2	7.7 ± 0.4	6.5 ± 0.3	7.1 ± 0.4	0.19	0.03^b^	0.99
2‐h glucose (mmol/L)	6.0 ± 0.3	6.8 ± 0.4	6.5 ± 0.3	6.8 ± 0.4	6.4 ± 0.3	6.9 ± 0.3	6.2 ± 0.3	6.6 ± 0.4	0.25	0.37	0.57
Basal insulin (mmol/L)	61.9 ± 4.2	57.4 ± 6.6	56.0 ± 5.4	47.6 ± 4.4	57.0 ± 6.5	49.2 ± 5.9	52.7 ± 4.7	62.3 ± 11.2	0.54	0.07	0.14
Mean insulin (mmol/L)	289.2 ± 30.4	343.0 ± 47.4	253.8 ± 36.0	360.4 ± 51.7	278.4 ± 28.5	344.7 ± 48.4	262.3 ± 39.0	299.5 ± 33.9	0.24	0.22	0.41

*Note*: All results are shown as mean ± SEM, *n* = 12 males, and *n* = 12 females. *p* values are listed in order as sex (S), trial (T) and sex × trial (S × T) determined using mixed model ANOVA.

^a^ Lower following LLHR versus CON.

^b^ Higher following MIC versus CON and LLHR.

There was no effect of sex (*p* = 0.18, *η*_p_^2^ = 0.08) or trial (*p* = 0.23, *η*_p_^2^ = 0.06, Figure 3h) on basal insulin clearance, however there was sex × trial interaction (*p* = 0.01 *η*_p_^2^ = 0.17) indicating that following MIC exercise females had a greater basal insulin clearance compared to males (Figure 3g). There was no effect of sex (*p* = 0.64, *η*_p_^2^ = 0.01) and no sex × trial interaction (*p* = 0.14, *η*_p_^2^ = 0.08) for insulin clearance during the OGTT, however, there was a significant main effect of trial (*p* = 0.02, *η*_p_^2^ = 0.14) indicating that insulin clearance during the OGTT was greater following HIIE compared to CON and MIC exercise (Figure 3g).

### 3.6 Measures of post‐exercise insulin sensitivity

**FIGURE 4:**
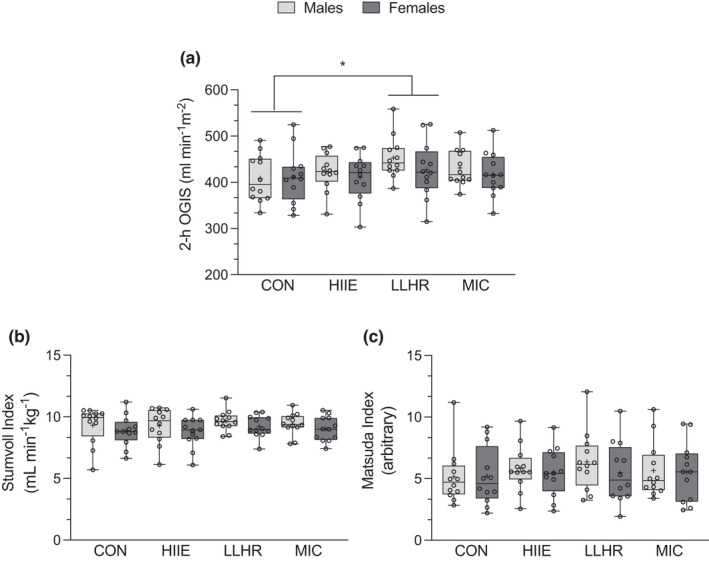
Insulin sensitivity indices in males (*n* = 12) and females (*n* = 12) at rest and following exercise (a) 2 h OGIS *higher following LLHR compared to CON determined from mixed model ANOVA (*p* = 0.005, *d* = 0.62); (b) Stumvoll insulin sensitivity index; (c) Matsuda insulin sensitivity index.

Measures of insulin sensitivity are found in Figure 4. There was no main effect of sex (*p* = 0.43, *η*_p_^2^ = 0.03) on 2‐h OGIS but there was a main effect of trial (*p* = 0.03, *η*_p_^2^ = 0.13) indicating that 2 h‐OGIS was significantly higher after LLHR compared to CON (Figure 4a). There was no significant trial × sex interaction (*p* = 0.74, *η*_p_^2^ = 0.03) for 2‐h OGIS. There was no effect of sex (*p* > 0.32, *η*_p_^2^ > 0.04), trial (*p* > 0.10, *η*_p_^2^ > 0.09) or sex × trial interaction (*p* > 0.50, *η*_p_^2^ > 0.01) for the Stumvoll or Matsuda insulin sensitivity indices (Figure 4b,c).

## 4 DISCUSSION

The purpose of the present study was to investigate the effects of sex and exercise mode on indices of pancreatic 𝛽‐cell function, insulin clearance, and insulin sensitivity in young, recreationally active males and females. We found no difference in basal 𝛽‐cell function, insulin clearance or insulin sensitivity in males and females matched for aerobic fitness. Furthermore, we found that exercise mode did not independently influence 𝛽‐cell function but did influence post‐exercise basal and mean glucose concentrations, insulin clearance, and insulin sensitivity. Specifically, following LLHR resistance exercise glucose concentration immediately prior to the OGTT was lower as compared with CON, mean glucose during the OGTT was lower versus MIC exercise and 2 h OGIS was higher as compared with CON. Additionally, following HIIE insulin clearance during the OGTT was higher as compared with CON and MIC exercise. Furthermore, while sex did not independently influence any of the measured outcomes, sex, and exercise mode interacted to influence glucose sensitivity and basal insulin clearance and tended to influence potentiation factor ratio. Specifically, in females glucose sensitivity was higher following LLHR and HIIE as compared with MIC exercise. Additionally, following MIC exercise basal insulin clearance was greater in females as compared with males and following HIIE potentiation factor ratio tended to be higher in males than females.

Understanding how 𝛽‐cell function changes with acute exercise is important in understanding how exercise influences glycemic control. Given that acute bouts of exercise have an immediate effect on insulin sensitivity (Sigal et al., 2004), it is possible that acute exercise may influence 𝛽‐cell function in a mode and/or intensity‐specific manner and thus, understanding whether different modes of exercise acutely influence 𝛽‐cell function can improve our understanding of the interplay between exercise and 𝛽‐cell function. In the present study, we found that exercise mode did not differentially influence markers of pancreatic 𝛽‐cell function in the study population as a whole. However, in females, only exercise mode influenced glucose sensitivity, the main parameter characterizing 𝛽‐cell function, with it being higher following LLHR and HIIE as compared with MIC exercise. Thus, our findings suggest that it is important to consider sex when examining how exercise influences 𝛽‐cell function and that different modes of exercise may differentially affect 𝛽‐cell function in females, which warrants further examination. Furthermore, our findings suggest, at least acutely in females, that LLHR and HIIE may be more efficacious than MIC exercise at improving 𝛽‐cell function. Training trials comparing the effectiveness of these bouts of exercise on 𝛽‐cell function with consideration for sex are needed in order to further our understanding of how different modes of exercise improve 𝛽‐cell function and insulin sensitivity and reduce the risk for T2D.

We also found that exercise mode influenced insulin clearance during the OGTT such that insulin clearance was greater following HIIE compared to CON and MIC exercise. Hyperinsulinemia impairs insulin receptor function, which overtime leads to insulin resistance and T2D (Kanety et al., 1994). Insulin clearance rates are lower in those who are obese and/or glucose intolerant and are a predictor of the development of T2D (Bojsen‐Moller et al., 2018; Piccinini & Bergman, 2020). Thus, enhanced insulin clearance may represent a key mechanism by which exercise reduces insulinemia and prevents the development of IR and T2D. Acute exercise has been found to increase insulin clearance as well as the activity of insulin‐degrading enzymes in liver and skeletal muscle (Kurauti, Costa‐Junior, et al., 2016; Kurauti, Freitas‐Dias, et al., 2016; Tuominen et al., 1997), however, to the best of our knowledge we are unaware of any trials that have compared the effects of different modes of exercise on insulin clearance. Our findings suggest that HIIE improves insulin clearance and is better than MIC exercise to promote post‐exercise insulin clearance, which may have implications for the mechanism by which different modes of exercise improve insulin sensitivity acutely and potentially following training as well. In fact, repeated enhanced post‐exercise insulin clearance may, at least in part, be one of the mechanisms by which high‐intensity interval training induces improvements in insulin sensitivity similar to that of moderate‐intensity continuous training despite the lower training volume (Little et al., 2014; Mitranun et al., 2014).

Similar to aerobic training, resistance training has also been found to improve indices of insulin sensitivity and 𝛽‐cell function (Croymans et al., 2013). In recent years, a novel form of resistance exercise, LLHR has been gaining popularity as it can elicit the same positive adaptations as traditional high‐load, low‐repetition resistance exercises with a much lower load (Fisher et al., 2017; Mitchell et al., 2012; Morton et al., 2016). A previous study reported that the combination of aerobic and resistance exercise induced greater improvements in 𝛽‐cell function than aerobic or resistance training alone (AbouAssi et al., 2015). However, the findings of this trial are complicated by the fact that the time spent training was greater in the combined training group than the aerobic or resistance training groups and thus the greater improvement seen may have been a result of greater training volume. In the current trial, we employed LLHR resistance exercise, which is a more aerobic form of resistance exercise due to its longer sets and less rest, in such a manner that we were able to directly compare the effects of ‘combined’ aerobic and resistance training to aerobic exercise of different intensities. As discussed above, in the current trial we found that an acute bout of LLHR had a greater effect on glucose sensitivity than MIC exercise, but this was only in females and not males. Furthermore, 2‐h OGIS was higher following LLHR, but not HIIE and MIC exercise, as compared with CON. Thus, our findings suggest that when matched for training time, the combination of aerobic and resistance exercise may be especially effective at improving 𝛽‐cell function and insulin action; however, training trials investigating the effects of LLHR are needed to fully ascertain the benefit of this mode of training. Furthermore, as noted above, given the sex difference in response observed in the current trial it will be vital that training trials consider sex in their study design.

Another main finding of the current study was the lack of sex difference in basal pancreatic 𝛽‐cell function between males and females. We did not find sex differences in parameters of 𝛽‐cell function such as basal or mean insulin during the OGTT, rate sensitivity or potentiation factor ratio at rest despite another study finding that during a standardized meal test healthy woman have greater postprandial plasma insulin and c‐peptide concentrations (Basu et al., 2006). An important difference between our trial and that of the aforementioned study is our trial investigated insulin and c‐peptide concentrations following an OGTT whereas they investigated insulin and c‐peptide concentrations following a mixed meal containing carbohydrates, protein, and fat, which may have resulted in a greater physiological response compared to the OGTT alone. Furthermore, when aerobic fitness was estimated using provided body composition data and measured V̇O_2_max, aerobic fitness relative to FFM was ~16% higher in males than females, suggesting that males were better trained than females, which may have influenced the findings. In our trial, we ensured that males and females were equally matched based on fitness relative to fat‐free mass which is also why we may not have seen a difference between the sexes in our study. The lack of sex difference in 𝛽‐cell function between males and females in the current trial is interesting because it is thought that estrogen may have a protective effect on pancreatic 𝛽‐cell function and survival (Louet et al., 2004). The protective effect of estrogen on 𝛽‐cell function is thought to be mainly due to estrogen receptor activation, which promotes survival and 𝛽‐cell mass expansion in rodents (Bernal‐Mizrachi et al., 2014) and may improve 𝛽‐cell mass in type 1 diabetes (Mauvais‐Jarvis, 2016). In the current trial, we tested females during the follicular phase of the menstrual cycle where estrogen is at its lowest concentration, thus, perhaps we did not find an effect of sex on 𝛽‐cell function due to the timing of testing. Future trials should examine whether 𝛽‐cell function differs between males and females in the luteal phase when estrogen concentrations are high. Furthermore, our sample included non‐diabetic healthy young males and females as we wanted to examine whether there were inherent sex differences in 𝛽‐cell function in the absence of pathology. Perhaps sex‐based differences become more apparent in pre‐diabetes or T2D with estrogen acting in a protective manner when the 𝛽‐cell is under stress. Thus, future trials should also examine basal differences in pancreatic 𝛽‐cell function between the sexes in those with insulin resistance while still considering aerobic fitness.

We also found differential effects of exercise between males and females. Specifically, despite there being no difference in basal insulin clearance between males and females during the CON OGTT, following MIC exercise basal insulin clearance was higher in females than males. Furthermore, while not significant, the potentiation factor ratio tended to be lower in females than males following HIIE. Insulin clearance and potentiation factor ratio both decrease as one moves from normal glucose tolerance towards T2D (Ferrannini et al., 2005; Mari et al., 2010; Piccinini & Bergman, 2020), thus our findings are suggestive that sex may influence which mode of exercise most benefits pancreatic function. The finding that potentiation factor ratio tended to be higher in males is in line with high‐intensity/sprint interval training trials that have found that insulin sensitivity improves in males, but not females (Gillen et al., 2014; Metcalfe et al., 2012). Future trials should examine whether sex influences the effect of different modes of exercise training on pancreatic 𝛽‐cell function in relation to the prevention of T2D.

A major strength of this trial is the proper matching of males and females for training status and controlled variation in sex hormones that may affect insulin action. It is often seen in research studies that males and females are not matched for fitness relative to fat‐free mass (V̇O_2peak_/kgFFM/min), but rather body weight, which could lead to inappropriate conclusions regarding inherent differences between the sexes and/or efficacy of acute bouts of exercise between the sexes due to baseline differences in fitness status. Furthermore, while it has been found that insulin sensitivity can vary across the menstrual cycle (Trout et al., 2007), whether pancreatic 𝛽‐cell function or c‐peptide concentration are affected by the menstrual cycle is largely unexamined (Lutoslawska et al., 2006), and whether exercise modifies the relationship between menstrual phase and pancreatic 𝛽‐cell function is unknown. As such, another strength of the current trial is that all females were tested during the mid‐follicular phase of the menstrual cycle to control for menstrual phase. Additionally, dietary intake was controlled throughout the study and participants ate similar meals before the CON OGTT and the post‐exercise OGTTs, thereby limiting any differences in insulin response or 𝛽‐cell function due to changes in dietary intake. A limitation to the current study is the use of the OGTT rather than the gold‐standard use of the hyperinsulinemic‐euglycemic clamp. The use of the hyperinsulinemic‐euglycemic clamp could potentially be more sensitive to slight changes in 𝛽‐cell function and response; however, the use of the clamp was not feasible in the current trial. However, while it would be interesting to use a hyperinsulinemic‐euglycemic clamp to examine how exercise and sex influence 𝛽‐cell function, due to the intravenous rather than oral route of glucose entry, the clamp is insensitive to the modifying effects of incretins on insulin secretion, which is an advantage of the use of the OGTT.

In conclusion, we found no differences in pancreatic 𝛽‐cell function at rest between males and females who did not differ in aerobic fitness. Additionally, while overall, we found that acute bouts of different modes of exercise largely had no effect on markers of pancreatic 𝛽‐cell function, we did find that glucose sensitivity was higher following HIIE and LLHR resistance exercise as compared with MIC exercise in females. Furthermore, we found that females had a significantly greater basal insulin clearance than males following MIC exercise and males tended to have a greater potentiation ratio than females following HIIE suggesting that sex and exercise mode interact to influence insulin clearance and 𝛽‐cell function. Moreover, exercise mode influenced insulin clearance and insulin sensitivity in both males and females. Our work highlights the need to consider biological sex when examining the effect of acute and chronic exercise on 𝛽‐cell function, insulin clearance, and insulin sensitivity. Future trials should examine if the physiological responses reported here in response to exercise in young, healthy males and females are upheld and/or more pronounced in individuals who are insulin resistant.

## AUTHOR CONTRIBUTIONS

Kayleigh M Beaudry and Michaela C Devries designed the research. Kayleigh M Beaudry conducted the research and sample collection. Kayleigh M Beaudry and Julian C Surdi performed sample analysis and analyzed the data. Andrea Mari performed all mathematical modeling calculations. Kayleigh M Beaudry drafted the manuscripts that was edited by Michaela C Devries. All authors edited and approved the final version of the manuscript. Kayleigh M Beaudry assumes final responsibility for the integrity of the data.

## FUNDING INFORMATION

Kayleigh M. Beaudry was supported by an Ontario Graduate Scholarship (OGS). Julian C. Surdi was supported by an NSERC Undergraduate Summer Research Award (USRA). This research was supported by a Natural Sciences and Engineering Research Council (NSERC) Discovery Grant to Michaela C. Devries.

## CONFLICT OF INTEREST

The authors declare no conflict of interest.

## References

[phy215354-bib-0001] AbouAssi, H. , Slentz, C. A. , Mikus, C. R. , Tanner, C. J. , Bateman, L. A. , Willis, L. H. , Shields, A. T. , Piner, L. W. , Penry, L. E. , Kraus, E. A. , Huffman, K. M. , Bales, C. W. , Houmard, J. A. , & Kraus, W. E. (2015). The effects of aerobic, resistance, and combination training on insulin sensitivity and secretion in overweight adults from STRRIDE AT/RT: A randomized trial. Journal of Applied Physiology (Bethesda, MD: 1985), 118(12), 1474–1482. 10.1152/japplphysiol.00509.2014 PMC4469920 25882384

[phy215354-bib-0022] Aguiree, F. , Brown, A. , Cho, N. H. , Dahlquist, G. , Dodd, S. , Dunning, T. , … Whiting, D. (2013). *IDF Diabetes Atlas*(6th).

[phy215354-bib-0002] Basu, R. , Dalla Man, C. , Campioni, M. , Basu, A. , Klee, G. , Toffolo, G. , … Rizza, R. A. (2006). Effects of age and sex on postprandial glucose metabolism: Differences in glucose turnover, insulin secretion, insulin action, and hepatic insulin extraction. Diabetes, 55(7), 2001–2014. 16804069 10.2337/db05-1692

[phy215354-bib-0003] Bernal‐Mizrachi, E. , Kulkarni, R. N. , Scott, D. K. , Mauvais‐Jarvis, F. , Stewart, A. F. , & Garcia‐Ocaña, A. (2014). Human β‐cell proliferation and intracellular signaling part 2: Still driving in the dark without a road map. Diabetes, 63(3), 819–831. 24556859 10.2337/db13-1146 PMC3931400

[phy215354-bib-0004] Bojsen‐Moller, K. N. , Lundsgaard, A. M. , Madsbad, S. , Kiens, B. , & Holst, J. J. (2018). Hepatic insulin clearance in regulation of systemic insulin concentrations‐role of carbohydrate and energy availability. Diabetes, 67(11), 2129–2136. 30348819 10.2337/db18-0539

[phy215354-bib-0005] Burd, N. A. , West, D. W. , Staples, A. W. , Atherton, P. J. , Baker, J. M. , Moore, D. R. , … Phillips, S. M. (2010). Low‐load high volume resistance exercise stimulates muscle protein synthesis more than high‐load low volume resistance exercise in young men. PLoS One, 5(8), e12033. 20711498 10.1371/journal.pone.0012033 PMC2918506

[phy215354-bib-0006] Canadian Institutes of Health Research , Natural Sciences and Engineering Research Council of Canada, & Social Sciences and Humanities Research Council of Canada. (2018). Tri‐Council Policy Statement: Ethical Conduct for Research Involving Humans.

[phy215354-bib-0007] Cohen, J. (1988). Statiscal power analysis for the behavioral sciences (2nd ed.). Lawrence Erlbaum Associates.

[phy215354-bib-0008] Croymans, D. M. , Paparisto, E. , Lee, M. M. , Brandt, N. , Le, B. K. , Lohan, D. , … Roberts, C. K. (2013). Resistance training improves indices of muscle insulin sensitivity and beta‐cell function in overweight/obese, sedentary young men. Journal of Applied Physiology (Bethesda, MD: 1985), 115(9), 1245–1253. 10.1152/japplphysiol.00485.2013 PMC3841835 23970530

[phy215354-bib-0009] Dela, F. , Ingersen, A. , Andersen, N. B. , Nielsen, M. B. , Petersen, H. H. H. , Hansen, C. N. , … Helge, J. W. (2019). Effects of one‐legged high‐intensity interval training on insulin‐mediated skeletal muscle glucose homeostasis in patients with type 2 diabetes. Acta Physiologica (Oxford, England), 226(2), e13245. 10.1111/apha.13245 30585698

[phy215354-bib-0010] Dela, F. , von Linstow, M. E. , Mikines, K. J. , & Galbo, H. (2004). Physical training may enhance beta‐cell function in type 2 diabetes. American Journal of Physiology. Endocrinology and Metabolism, 287(5), E1024–E1031. 15251867 10.1152/ajpendo.00056.2004

[phy215354-bib-0011] Devries, M. C. , Hamadeh, M. J. , Phillips, S. M. , & Tarnopolsky, M. A. (2006). Menstrual cycle phase and sex influence muscle glycogen utilization and glucose turnover during moderate‐intensity endurance exercise. American Journal of Physiology. Regulatory, Integrative and Comparative Physiology, 291(4), R1120–R1128. 16690766 10.1152/ajpregu.00700.2005

[phy215354-bib-0012] DiPietro, L. , Dziura, J. , Yeckel, C. W. , & Neufer, P. D. (2006). Exercise and improved insulin sensitivity in older women: evidence of the enduring benefits of higher intensity training. Journal of Applied Physiology (Bethesda, MD: 1985), 100(1), 142–149. 10.1152/japplphysiol.00474.2005 16141382

[phy215354-bib-0013] Ferguson, C. J. (2009). An effect size primer: A guide for clinicians and researchers. Professional Psychology: Research and Practice, 40(5), 532–538.

[phy215354-bib-0014] Ferrannini, E. , Gastaldelli, A. , Miyazaki, Y. , Matsuda, M. , Mari, A. , & DeFronzo, R. A. (2005). beta‐Cell function in subjects spanning the range from normal glucose tolerance to overt diabetes: a new analysis. The Journal of Clinical Endocrinology and Metabolism, 90(1), 493–500. 15483086 10.1210/jc.2004-1133

[phy215354-bib-0015] Fisher, J. , Steele, J. , & Smith, D. (2017). High‐ and low‐load resistance training: Interpretation and practical application of current research findings. Sports Medicine, 47(3), 393–400. 27480764 10.1007/s40279-016-0602-1

[phy215354-bib-0016] Gannon, M. , Kulkarni, R. N. , Tse, H. M. , & Mauvais‐Jarvis, F. (2018). Sex differences underlying pancreatic islet biology and its dysfunction. Molecular Metabolism, 15, 82–91. 29891438 10.1016/j.molmet.2018.05.017 PMC6066785

[phy215354-bib-0017] Gibala, M. J. , Gillen, J. B. , & Percival, M. E. (2014). Physiological and health‐related adaptations to low‐volume interval training: Influences of nutrition and sex. Sports Medicine, 44(Suppl 2), S127–S137. 25355187 10.1007/s40279-014-0259-6 PMC4213388

[phy215354-bib-0018] Gillen, J. B. , Percival, M. E. , Skelly, L. E. , Martin, B. J. , Tan, R. B. , Tarnopolsky, M. A. , & Gibala, M. J. (2014). Three minutes of all‐out intermittent exercise per week increases skeletal muscle oxidative capacity and improves cardiometabolic health. PLoS One, 9(11), e111489. 25365337 10.1371/journal.pone.0111489 PMC4218754

[phy215354-bib-0020] Hood, M. S. , Little, J. P. , Tarnopolsky, M. A. , Myslik, F. , & Gibala, M. J. (2011). Low‐volume interval training improves muscle oxidative capacity in sedentary adults. Medicine and Science in Sports and Exercise, 43(10), 1849–1856. 21448086 10.1249/MSS.0b013e3182199834

[phy215354-bib-0021] Horie, I. , Abiru, N. , Eto, M. , Sako, A. , Akeshima, J. , Nakao, T. , … Kawakami, A. (2018). Sex differences in insulin and glucagon responses for glucose homeostasis in young healthy Japanese adults. Journal of Diabetes Investigation, 9(6), 1283–1287. 29489067 10.1111/jdi.12829 PMC6215950

[phy215354-bib-0023] Kanety, H. , Moshe, S. , Shafrir, E. , Lunenfeld, B. , & Karasik, A. (1994). Hyperinsulinemia induces a reversible impairment in insulin receptor function leading to diabetes in the sand rat model of non‐insulin‐dependent diabetes mellitus. Proceedings of the National Academy of Sciences of the United States of America, 91(5), 1853–1857. 8127894 10.1073/pnas.91.5.1853 PMC43262

[phy215354-bib-0024] Kurauti, M. A. , Costa‐Junior, J. M. , Ferreira, S. M. , Dos Santos, G. J. , Protzek, A. O. , Nardelli, T. R. , … Boschero, A. C. (2016). Acute exercise restores insulin clearance in diet‐induced obese mice. Journal of Endocrinology, 229(3), 221–232. 27000684 10.1530/JOE-15-0483

[phy215354-bib-0025] Kurauti, M. A. , Freitas‐Dias, R. , Ferreira, S. M. , Vettorazzi, J. F. , Nardelli, T. R. , Araujo, H. N. , … Costa‐Junior, J. M. (2016). Acute exercise improves insulin clearance and increases the expression of insulin‐degrading enzyme in the liver and skeletal muscle of swiss mice. PLoS One, 11(7), e0160239. 27467214 10.1371/journal.pone.0160239 PMC4965115

[phy215354-bib-0026] Lauritzen, H. P. (2013). Insulin‐ and contraction‐induced glucose transporter 4 traffic in muscle: Insights from a novel imaging approach. Exercise and Sport Sciences Reviews, 41(2), 77–86. 23072821 10.1097/JES.0b013e318275574c PMC3602324

[phy215354-bib-0027] Little, J. P. , Jung, M. E. , Wright, A. E. , Wright, W. , & Manders, R. J. (2014). Effects of high‐intensity interval exercise versus continuous moderate‐intensity exercise on postprandial glycemic control assessed by continuous glucose monitoring in obese adults. Applied Physiology, Nutrition, and Metabolism, 39(7), 835–841. 10.1139/apnm-2013-0512 24773254

[phy215354-bib-0028] Louet, J. F. , LeMay, C. , & Mauvais‐Jarvis, F. (2004). Antidiabetic actions of estrogen: Insight from human and genetic mouse models. Current Atherosclerosis Reports, 6(3), 180–185. 15068742 10.1007/s11883-004-0030-9

[phy215354-bib-0029] Lutoslawska, G. , Niedbalska, M. , Skierska, E. , Keska, A. , Szpocinska‐Byszewska, E. , & Zołnowska, M. (2006). Plasma proinsulin, C‐peptide and sex hormone concentrations in regularly menstruating premenopausal females with ovulatory and anovulatory menstrual cycles. The Journal of Sports Medicine and Physical Fitness, 46(1), 138–142. 16596113

[phy215354-bib-0030] Madsen, S. M. , Thorup, A. C. , Overgaard, K. , & Jeppesen, P. B. (2015). High intensity interval training improves glycaemic control and pancreatic beta cell function of type 2 diabetes patients. PLoS One, 10(8), e0133286. 26258597 10.1371/journal.pone.0133286 PMC4530878

[phy215354-bib-0031] Malin, S. K. , Francois, M. E. , Eichner, N. Z. M. , Gilbertson, N. M. , Heiston, E. M. , Fabris, C. , & Breton, M. (2018). Impact of short‐term exercise training intensity on β‐cell function in older obese adults with prediabetes. Journal of Applied Physiology (Bethesda, MD: 1985), 125(6), 1979–1986. 10.1152/japplphysiol.00680.2018 PMC6842889 30307821

[phy215354-bib-0032] Malin, S. K. , Hinnerichs, K. R. , Echtenkamp, B. G. , Evetovich, T. K. , & Engebretsen, B. J. (2013). Effect of adiposity on insulin action after acute and chronic resistance exercise in non‐diabetic women. European Journal of Applied Physiology, 113(12), 2933–2941. 24072034 10.1007/s00421-013-2725-5

[phy215354-bib-0033] Malin, S. K. , Rynders, C. A. , Weltman, J. Y. , Barrett, E. J. , & Weltman, A. (2016). Exercise intensity modulates glucose‐stimulated insulin secretion when adjusted for adipose, liver and skeletal muscle insulin resistance. PLoS One, 11(4), e0154063. 27111219 10.1371/journal.pone.0154063 PMC4844153

[phy215354-bib-0034] Mann, S. , Beedie, C. , Balducci, S. , Zanuso, S. , Allgrove, J. , Bertiato, F. , & Jimenez, A. (2014). Changes in insulin sensitivity in response to different modalities of exercise: a review of the evidence. Diabetes/Metabolism Research and Reviews, 30(4), 257–268. 24130081 10.1002/dmrr.2488

[phy215354-bib-0035] Mari, A. , Bagger, J. I. , Ferrannini, E. , Holst, J. J. , Knop, F. K. , & Vilsboll, T. (2013). Mechanisms of the incretin effect in subjects with normal glucose tolerance and patients with type 2 diabetes. PLoS One, 8(9), e73154. 24019903 10.1371/journal.pone.0073154 PMC3760909

[phy215354-bib-0036] Mari, A. , Pacini, G. , Murphy, E. , Ludvik, B. , & Nolan, J. J. (2001). A model‐based method for assessing insulin sensitivity from the oral glucose tolerance test. Diabetes Care, 24(3), 539–548. 11289482 10.2337/diacare.24.3.539

[phy215354-bib-0037] Mari, A. , Tura, A. , Natali, A. , Laville, M. , Laakso, M. , Gabriel, R. , … Ferrannini, E. (2010). Impaired beta cell glucose sensitivity rather than inadequate compensation for insulin resistance is the dominant defect in glucose intolerance. Diabetologia, 53(4), 749–756. 20225397 10.1007/s00125-009-1647-6

[phy215354-bib-0038] Matsuda, M. , & DeFronzo, R. A. (1999). Insulin sensitivity indices obtained from oral glucose tolerance testing; comparsion with the euglycemic insulin clamp. Diabetes Care, 22(9), 1462–1470. 10480510 10.2337/diacare.22.9.1462

[phy215354-bib-0039] Mauvais‐Jarvis, F. (2016). Role of sex steroids in beta cell function, growth, and survival. Trends in Endocrinology and Metabolism, 27(12), 844–855. 27640750 10.1016/j.tem.2016.08.008 PMC5116277

[phy215354-bib-0040] Metcalfe, R. S. , Babraj, J. A. , Fawkner, S. G. , & Vollaard, N. B. (2012). Towards the minimal amount of exercise for improving metabolic health: Beneficial effects of reduced‐exertion high‐intensity interval training. European Journal of Applied Physiology, 112(7), 2767–2775. 22124524 10.1007/s00421-011-2254-z

[phy215354-bib-0041] Mitchell, C. J. , Churchward‐Venne, T. A. , West, D. W. D. , Burd, N. A. , Breen, L. , Baker, S. K. , & Phillips, S. M. (2012). Resistance exercise load does not determine training‐mediated hypertrophic gains in young men. Journal of Applied Physiology, 113(1), 71–77. 22518835 10.1152/japplphysiol.00307.2012 PMC3404827

[phy215354-bib-0042] Mitranun, W. , Deerochanawong, C. , Tanaka, H. , & Suksom, D. (2014). Continuous vs interval training on glycemic control and macro‐ and microvascular reactivity in type 2 diabetic patients. Scandinavian Journal of Medicine & Science in Sports, 24(2), e69–e76. 24102912 10.1111/sms.12112

[phy215354-bib-0043] Morton, R. W. , Oikawa, S. Y. , Wavell, C. G. , Mazara, N. , McGlory, C. , Quadrilatero, J. , … Phillips, S. M. (2016). Neither load nor systemic hormones determine resistance training‐mediated hypertrophy or strength gains in resistance‐trained young men. Journal of Applied Physiology (Bethesda, MD: 1985), 121(1), 129–138. 10.1152/japplphysiol.00154.2016 PMC4967245 27174923

[phy215354-bib-0044] Nuutila, P. , Knuuti, M. J. , Mäki, M. , Laine, H. , Ruotsalainen, U. , Teräs, M. , … Yki‐Järvinen, H. (1995). Gender and insulin sensitivity in the heart and in skeletal muscles: Studies using positron emission tomography. Diabetes, 44(1), 31–36. 7813811 10.2337/diab.44.1.31

[phy215354-bib-0045] Palmu, S. , Kuneinen, S. , Kautiainen, H. , Eriksson, J. G. , & Korhonen, P. E. (2021). Body surface area may explain sex differences in findings from the oral glucose tolerance test among subjects with normal glucose tolerance. Nutrition, Metabolism, and Cardiovascular Diseases, 31(9), 2678–2684. 10.1016/j.numecd.2021.05.018 34218989

[phy215354-bib-0046] Patarrão, R. S. , Wayne Lautt, W. , & Paula Macedo, M. (2014). Assessment of methods and indexes of insulin sensitivity. Revista Portuguesa de Endocrinologia, Diabetes e Metabolismo, 9(1), 65–73.

[phy215354-bib-0047] Piccinini, F. , & Bergman, R. N. (2020). The measurement of insulin clearance. Diabetes Care, 43(9), 2296–2302. 32910777 10.2337/dc20-0750 PMC7440908

[phy215354-bib-0048] Pomerleau, J. , McKeigue, P. M. , & Chaturvedi, N. (1999). Relationships of fasting and postload glucose levels to sex and alcohol consumption. Are American Diabetes Association criteria biased against detection of diabetes in women? Diabetes Care, 22(3), 430–433. 10097924 10.2337/diacare.22.3.430

[phy215354-bib-0019] Roglic, G. (Ed.). (2016). Global Report on Diabetes. World Health Organization.

[phy215354-bib-0049] Schoenfeld, B. J. , Peterson, M. D. , Ogborn, D. , Contreras, B. , & Sonmez, G. T. (2015). Effects of Low‐ vs. High‐load resistance training on muscle strength and hypertrophy in well‐trained men. Journal of Strength and Conditioning Research, 29(10), 2954–2963. 25853914 10.1519/JSC.0000000000000958

[phy215354-bib-0050] Sicree, R. A. , Zimmet, P. Z. , Dunstan, D. W. , Cameron, A. J. , Welborn, T. A. , & Shaw, J. E. (2008). Differences in height explain gender differences in the response to the oral glucose tolerance test‐ the AusDiab study. Diabetic Medicine, 25(3), 296–302. 18307457 10.1111/j.1464-5491.2007.02362.x

[phy215354-bib-0051] Sigal, R. J. , Kenny, G. P. , Wasserman, D. H. , & Castaneda‐Sceppa, C. (2004). Physical activity/exercise and type 2 diabetes. Diabetes Care, 27(10), 2518–2539. 15451933 10.2337/diacare.27.10.2518

[phy215354-bib-0052] Slentz, C. A. , Tanner, C. J. , Bateman, L. A. , Durheim, M. T. , Huffman, K. M. , Houmard, J. A. , & Kraus, W. E. (2009). Effects of exercise training intensity on pancreatic beta‐cell function. Diabetes Care, 32(10), 1807–1811. 19592624 10.2337/dc09-0032 PMC2752909

[phy215354-bib-0053] Sogaard, D. , Lund, M. T. , Scheuer, C. M. , Dehlbaek, M. S. , Dideriksen, S. G. , Abildskov, C. V. , … Helge, J. W. (2017). High‐intensity interval training improves insulin sensitivity in older individuals. Acta Physiologica (Oxford, England), 222(4), e13009. 10.1111/apha.13009 29197155

[phy215354-bib-0054] Stumvoll, M. , Mitrakou, A. , Pimenta, W. , Jenssen, T. , Yki‐Jarvinen, H. , Van Haeften, T. , … Gerich, J. (2000). Use of the oral glucose tolerance test to assess insulin release and insulin sensitivity. Diabetes Care, 23(3), 295–301. 10868854 10.2337/diacare.23.3.295

[phy215354-bib-0055] Tarnopolsky, M. A. (2008). Sex differences in exercise metabolism and the role of 17‐beta estradiol. Medicine and Science in Sports and Exercise, 40(4), 648–654. 18317381 10.1249/MSS.0b013e31816212ff

[phy215354-bib-0056] Tessier, D. , Menard, J. , Fulop, T. , Ardilouze, J. , Roy, M. , Dubuc, N. , … Gauthier, P. (2000). Effects of aerobic physical exercise in the elderly with type 2 diabetes mellitus. Archives of Gerontology and Geriatrics, 31(2), 121–132. 11090907 10.1016/s0167-4943(00)00076-5

[phy215354-bib-0057] Trout, K. K. , Rickels, M. R. , Schutta, M. H. , Petrova, M. , Freeman, E. W. , Tkacs, N. C. , & Teff, K. L. (2007). Menstrual cycle effects on insulin sensitivity in women with type 1 diabetes: a pilot study. Diabetes Technology & Therapeutics, 9(2), 176–182. 17425444 10.1089/dia.2006.0004

[phy215354-bib-0058] Tuominen, J. A. , Ebeling, P. , & Koivisto, V. A. (1997). Exercise increases insulin clearance in healthy man and insulin‐dependent diabetes mellitus patients. Clinical Physiology, 17(1), 19–30. 9015655 10.1046/j.1365-2281.1997.01717.x

[phy215354-bib-0059] Utzschneider, K. M. , Prigeon, R. L. , Tong, J. , Gerchman, F. , Carr, D. B. , Zraika, S. , … Kahn, S. E. (2007). Within‐subject variability of measures of beta cell function derived from a 2 h OGTT: Implications for research studies. Diabetologia, 50(12), 2516–2525. 17928990 10.1007/s00125-007-0819-5

[phy215354-bib-0060] Varlamov, O. , Bethea, C. L. , & Roberts, C. T., Jr. (2014). Sex‐specific differences in lipid and glucose metabolism. Frontiers in Endocrinology, 5, 241. 25646091 10.3389/fendo.2014.00241 PMC4298229

[phy215354-bib-0061] Yang, Z. , Scott, C. A. , Mao, C. , Tang, J. , & Farmer, A. J. (2014). Resistance exercise versus aerobic exercise for type 2 diabetes: A systematic review and meta‐analysis. Sports Medicine, 44(4), 487–499. 24297743 10.1007/s40279-013-0128-8

